# Communications Received

**Published:** 1873-09

**Authors:** 


*Communications Received.*

The editors of the RECORD take great pleasure in acknowledging the reception of the following original papers, which shall appear, as soon as possible, in its pages. They cordially invite communications from professional gentlemen of ALL SECTIONS, and open the columns of the RECORD to MEDICAL SOCIETIES and ASSOCIATIONS, as well as the communications of their members, EVERY WHERE.

Ergot as an Antihemorrhagic, by T. Curtis Smith, M.D., of Ohio; Puerperal Fever, by J. G. Knox, M.D., of Mississippi; Metro-Peritonitis, with Abdominal Abscess, Successfully Opened in the Linea Alba, by A. R. Kilpatrick, M.D., of Texas; Pulmonary Emphysema, its Causes, Symptoms and Treatment, by C. H. Tidd, M.D., of Ohio (for which we thank Dr. T. Curtis Smith;) Vitality and its Co-ordinate Forces, by W. T. Grant, M.D., of Georgia; Salicine in Colliquative Diarrhœa, by J. D. Tucker, M. D., of Tennessee; Clinical History of a Case of Diphtheritic Metritis, and one of Acute Diabetes, by John E. Lockridge, M.D., of Virginia; Remedy for Tapeworm, by W. W. Harrison, M.D., of Alabama; Report of a Case, by A. S. Helmick, M.D., of Louisiana; Poisoning by Strychnia, by E. T. Henry, M.D., of Miss.

We assure our friend, Dr. J. A. Alexander, that we will take great pleasure in publishing, at an early day, the useful and scientific article of Dr. J. W. Smith, which he so kindly furnished us.

The readers of the RECORD will perceive that the kindness of its friends will *force* its enlargement, which we promise to do in January next, if they will, in the *proper manner*, second our efforts to give them a *wide-awake* journal. Please "take due notice and govern yourselves accordingly."

